# Kyphoplasty Solves Stabilization and Pain Control Inadequately Treated via Prior Hartshill Rectangle for Thoracolumbar Burst Fracture

**DOI:** 10.7759/cureus.18525

**Published:** 2021-10-06

**Authors:** Omron Hassan, Tapasya Surti, Scott G Glickman

**Affiliations:** 1 Department of Clinical Sciences, Touro University Nevada College of Osteopathic Medicine, Henderson, USA; 2 Department of Neurosurgery, Center for Neurosurgery Las Vegas, Las Vegas, USA

**Keywords:** hartshill rectangle, burst fracture, kyphoplasty, minimally invasive, percutaneous, spine surgery, thoracolumbar

## Abstract

Surgical management of spinal burst fractures has progressed to include minimally invasive techniques as preferred modalities of treatment. Burst fractures with indications for surgical treatment either through instability or intractable pain classically have required pedicle screw fixation, which requires extensive dissection resulting in postoperative pain and significant recovery time, and also requires longer operative times with more potential blood loss. Balloon kyphoplasty is an established percutaneous technique that can provide quick pain relief for patients with intractable pain following compression and burst fractures, and vertebral body height can also be restored. In the present case, a female patient was seen in the emergency room with intractable pain and a dehiscent thoracolumbar incision after recently undergoing surgery with placement of a Hartshill rectangle and sublaminar wires at another institution for a T12 burst fracture (AO classification [AO] A4 and thoracolumbar injury classification and severity score [TLICS] 4) caused by a motor vehicle accident. Imaging identified an acute unhealed fracture at T12 and other vertebrae with questionable lesions. She underwent surgery to remove the Hartshill construct, stabilize the fracture, biopsy lesions (T7, T10, L2, and L4), and debride and close the wound. Following hardware removal, kyphoplasty was then performed through the open exposure at T12, which could have otherwise been done percutaneously. The patient experienced immediate and complete resolution of her pain associated with the fracture and had no neurological deficits. Modern minimally invasive techniques including kyphoplasty should be favored when indicated as alternative treatment options over more invasive treatment modalities, as they lead to quicker resolution of pain and recovery when compared to techniques requiring a large exposure.

## Introduction

Spinal burst fractures occur during high-impact trauma from compressive forces and also develop progressively in the face of osteoporosis or malignancy. Immobilization via spinal bracing along with pain medications is often the initial non-invasive form of treatment. Recovery often takes 8 to 12 weeks or longer and a well-fitted orthosis increases treatment success if the patient is compliant [1]. Surgical treatment of burst fractures is frequently warranted for fracture reduction and stabilization with indications including significant vertebral kyphotic deformity, spinal canal compromise, neurological deficit, and intractable pain [1]. If left untreated, unstable burst fractures may lead to ongoing bone pain, radiculopathy, and significant neurological deficits including spinal cord compromise.

Commonly employed surgical options for the treatment of burst fractures often include screw fixation via a posterior approach. However, further surgical techniques involve combined anterior/posterior fixation, corpectomy and reconstruction, and simple laminectomy, although the latter may lead to further destabilization. There are minimally invasive surgery (MIS) options such as percutaneous screw fixation, lateral corpectomy, and kyphoplasty [2]. Kyphoplasty is the least invasive and most muscle-sparing treatment option, which often along with correction of the neurological deficit through restoring vertebral body height, leads to the immediate resolution of pain postoperatively [3].

The Hartshill rectangle is a spinal fixation device occasionally used in the 1980s to treat burst fractures or spine deformity through the use of a single rectangular closed-loop metal rod and sublaminar wires and was thought to be especially beneficial in osteoporotic patients due to its high pull-out strength [4,5]. Hartshill rectangle with sublaminar wires have a high failure rate and a retrospective analysis by Ward et al. found 19 out of 43 patients to have unsatisfactory stabilization regardless of the number of involved columns [6]. The Hartshill rectangle is now considered an unnecessarily invasive treatment option in the United States for uncomplicated or simple burst fractures, especially with the availability of superior techniques.

We present a case of a patient who underwent recent Hartshill rectangle fixation with sublaminar wires to treat a traumatic burst fracture after a motor vehicle accident and without improvement in either pain or healing of her fracture. Revision involved removal of the Hartshill construct with successful resolution of the patient’s intractable pain and fracture stabilization via simple kyphoplasty.

## Case presentation

**Figure 1:**
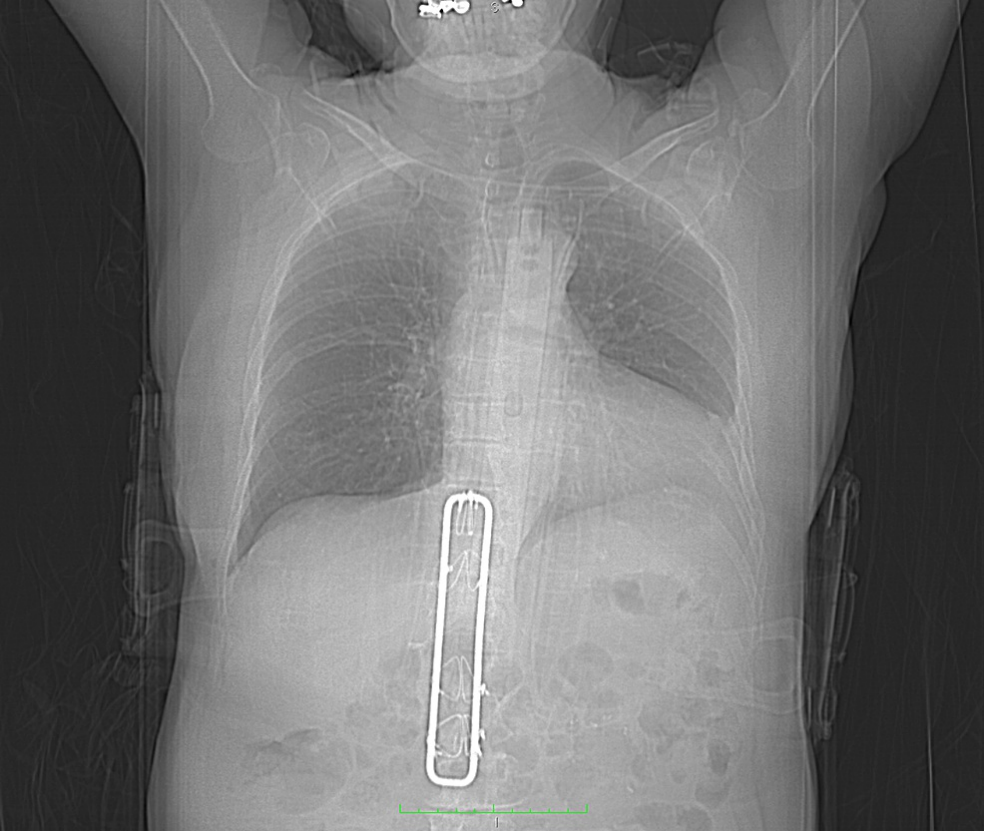
Preoperative X-ray Preoperative X-ray identifying Hartshill rectangle and sublaminar wires.

A 75-year-old woman presented to the emergency department with intractable back pain. Less than one month prior, she was involved in a motor vehicle accident and underwent placement of a Hartshill rectangle and sublaminar wires at a trauma center in a major metropolitan area in the western US to treat a T12 burst fracture. The patient arrived at our institution still wearing a thoracic lumbar sacral orthosis brace and physical examination revealed a bandaged, dehiscent, and draining surgical wound on her back. She had no neurological deficits, but an elevation of the bed to 30 degrees or more gave the patient further discomfort, and she had difficulty rotating in bed due to her severe pain. X-ray of the lumbar region identified a rectangular structure with sublaminar wires identified as a Hartshill rectangle (Figure 1).

**Figure 2:**
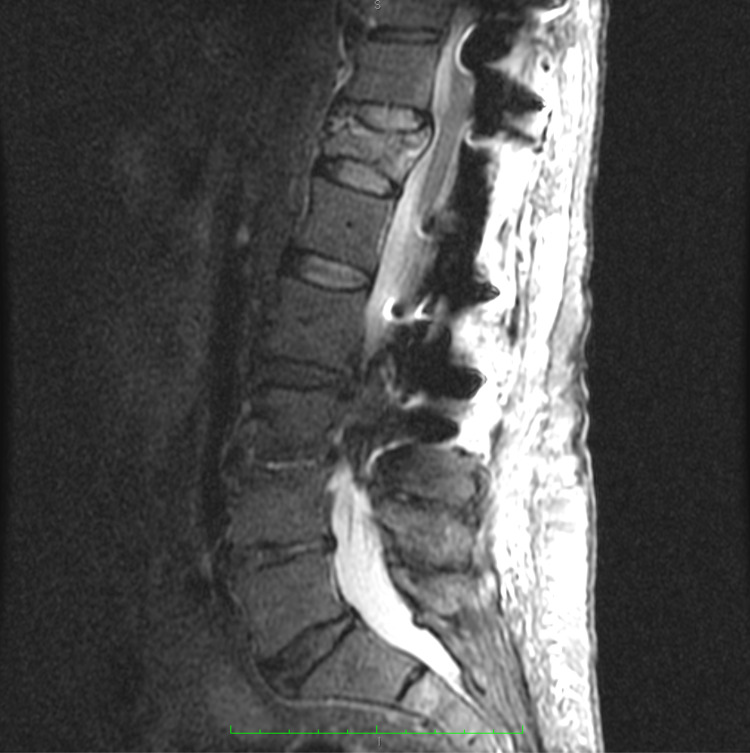
Preoperative STIR T2-weighted MRI Preoperative STIR T2-weighted MRI identifying acute to subacute burst fracture at T12 and artifact from the previously implanted hardware. STIR, short TI inversion recovery.

The burst fracture (AO classification [AO] A4 and thoracolumbar injury classification and severity score [TLICS] 4) was characterized on a short TI inversion recovery (STIR) sequence T2-weighted magnetic resonance imaging (MRI) scan as acute-appearing (Figure 2).

There were also notable vertebral body “lesions” at T7, T10, L2, and L4, which were all suspicious for metastatic disease or infection associated with a right suprarenal mass. The patient had no previous diagnosis of malignancy. No diagnostic testing or biopsy had been performed during the original hospitalization or as an outpatient. Due to the patient's signs and symptoms, imaging findings, and clinical condition, including wound-related issues and intractable pain, as well as unclear etiology of the bony abnormalities and the recently identified suprarenal mass, optimal clinical course included removal of the Hartshill rectangle and kyphoplasty at T12, L2, and L4, and percutaneous needle biopsy with radiofrequency ablation at T7, T10, L2, and L4, to treat the patient's pain, restore vertebral body height, collect cultures, and obtain biopsies of bony abnormalities suspected to be metastatic lesions.

**Figure 3:**
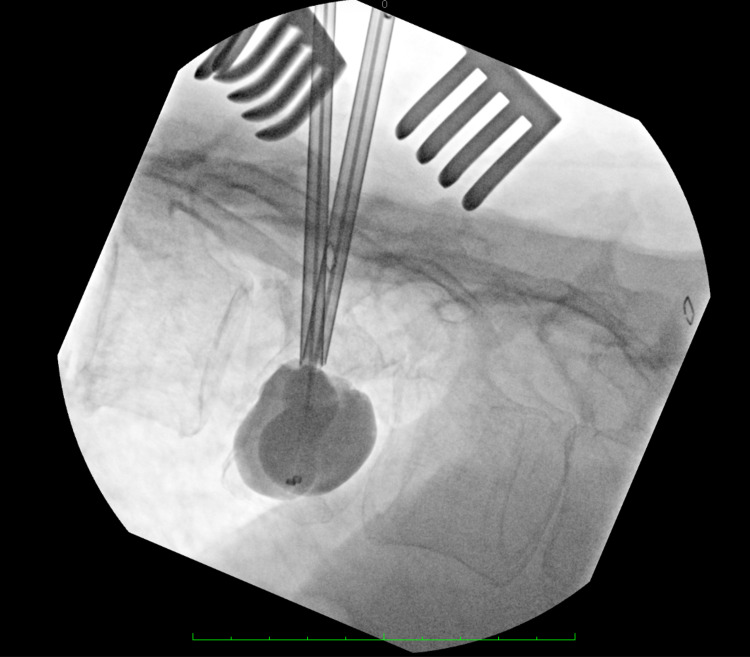
Intraoperative contrast injection into T12 Intraoperative fluoroscopy image showing Jamshidi needle positioning and contrast injection into T12, with kyphoplasty cement augmentation to follow.

The patient was taken to the operating room and placed prone along with anteroposterior (AP) and lateral C-arms that were positioned for intraoperative imaging. There was a 24 cm grossly dehiscent thoracolumbar wound that was reopened and sharply debrided and wound cultures were taken. The intact Hartshill construct with sublaminar wires was identified and removed and cultures were taken of the hardware. There was no evidence of bone graft used along with the Hartshill construct. Core needle biopsies were then taken at the suspected metastatic levels using a Jamshidi needle, followed by radiofrequency ablation of all biopsy points. Kyphoplasty was subsequently performed by first injecting contrast into each level bilaterally through transpedicular Jamshidi needles followed by bone cement under fluoroscopic guidance (Figure 3).

The patient was stable after the procedure and did not encounter any intraoperative complications.

**Figure 4:**
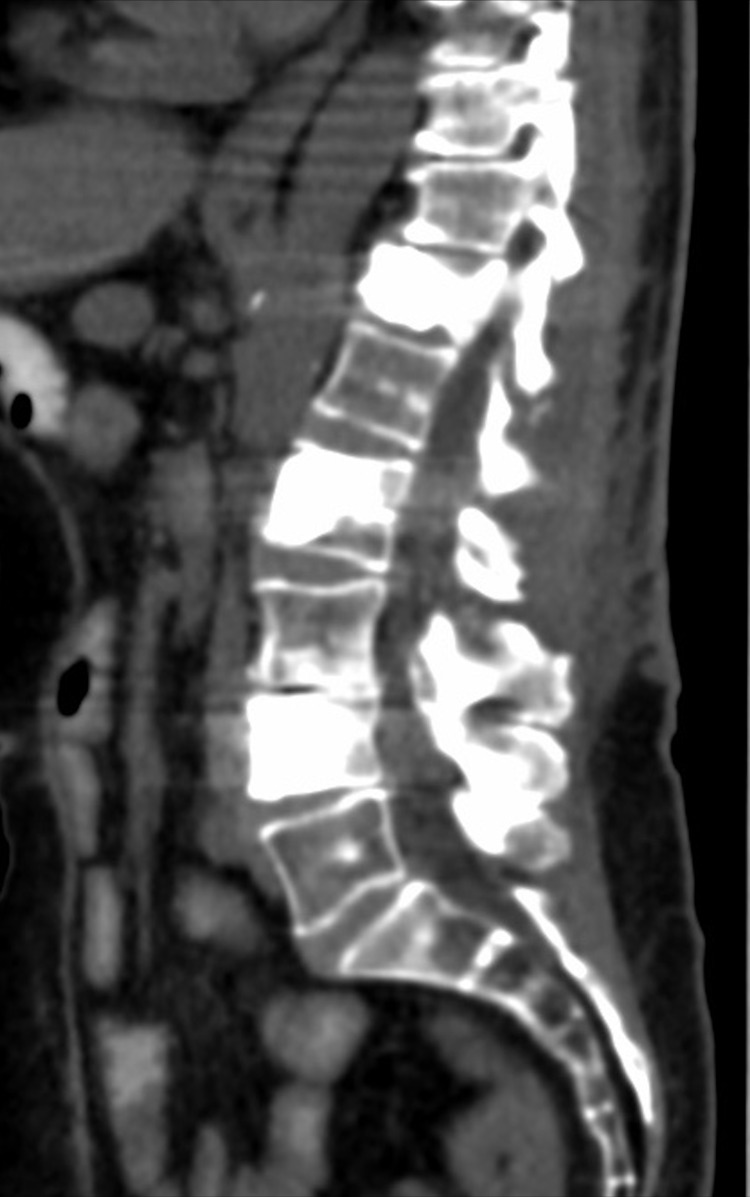
Postoperative CT at three-month follow-up Postoperative CT status post removal of hardware and kyphoplasty at T12 during three-month follow-up.

Upon the next day following surgery, the patient’s initial pain was completely resolved with no neurological deficits. The results of the intraoperative cultures did not reveal any infectious pathogens and all came back as negative. At one-month follow-up, the patient reported her pain to be completely resolved and without neurologic deficits. At three-month follow-up, the patient continued to have symptom relief and a computed tomography (CT) scan demonstrated maintained restoration of vertebral body height at T12 as well as L2 and L4 (Figure 4).

## Discussion

Treatment options for burst fractures continue to grow and when minimally invasive alternatives to open surgical procedures are available and indicated, they may result in quicker recovery times, shorter operative times, and decreased blood loss while still achieving adequate surgical correction such as restoration of vertebral body height seen with kyphoplasty. This patient suffered a persistent post-traumatic burst fracture without canal involvement or other spinal injuries (AO A4, TLICS 4) and needed correction due to her intractable pain, which would have been more effectively treated percutaneously with simple kyphoplasty. However, she underwent an unnecessarily invasive and ineffective Hartshill procedure without graft application despite being treated at a modern facility trauma center in the US, which had access to up-to-date equipment and treatment standards. We demonstrate here successful treatment of the intractable pain associated with a burst fracture and restoration of vertebral body height, and although the kyphoplasty was performed in an open fashion, this patient could have undergone percutaneous kyphoplasty initially after her injury rather than placement of a Hartshill rectangle and may have avoided unnecessary pain and reoperation.

There are evolving standards of care for the treatment of spinal burst fractures [7,8]. Establishing suggested treatments for such cases is based on the severity of fractures, levels of involvement, and combined pathology. Kyphoplasty is a viable MIS technical approach to burst fractures as it can be performed percutaneously while restoring vertebral body height and allowing kyphosis correction [3]. In comparison to other commonly applied techniques for treating burst fractures including posterior screw fixation, kyphoplasty has shown similar rates of pain reduction and stability while reducing muscle injury that would otherwise occur from dissection during exposure, which has also allowed for earlier mobilization in patients [8]. A recent comparison of kyphoplasty against pedicle screw fixation combined with cement augmentation demonstrated similar 30-day morbidity between the two techniques and both achieved significant early postoperative radiographic improvements; however, kyphoplasty resulted in significantly shorter surgical times, length of stay, reduction in opioid usage after discharge from the hospital, and similar clinical outcomes three months following surgery [9]. Furthermore, biomechanical stability of vertebral bodies post-kyphoplasty based on strain measurements is also comparable to intact vertebral bodies, which may contribute to successful clinical outcomes seen with the technique [10].

## Conclusions

The debate over the standard of care for the treatment of acute and subacute burst fractures is ongoing, and careful consideration of the patient’s history and severity of fracture should be used to determine an appropriate technical approach whether it be through an open or MIS technique. MIS techniques should be favored over open techniques when possible, and the Hartshill rectangle technique has little to no further benefit in current treatment for compression fractures and should be abandoned. Additionally, treatment of traumatic burst fractures should be performed by surgeons trained in all options of care ranging from percutaneous, hybrid, and open techniques to ensure the best delivery of care.
